# Navigating Emergency Care After Injury: A Qualitative Study of Asylum Seekers and Refugees in England and Wales Using the Candidacy Framework (BE SURE Study)

**DOI:** 10.1111/hex.70861

**Published:** 2026-09-11

**Authors:** Ashrafunnesa Khanom, Lydia Jones, Hanifah M. Khanom, Fadi Baghdadi, Ann John, Alison Porter, Helen Snooks, Niroshan Siriwardena, Julia Williams, Thanuja Hettiarachchi, Solmaz Safari

**Affiliations:** ^1^ Swansea University Medical School, Swansea University Swansea UK; ^2^ Community and Health Research Unit (CaHRU), University of Lincoln Lincoln UK; ^3^ Centre for Applied Clinical, Health and Care Research (CACHE) School of Health and Social Work, University of Hertfordshire Hertfordshire UK

**Keywords:** asylum seekers, delivery of healthcare, emergency, health personnel, pre‐hospital, qualitative research, refugees

## Abstract

**Background:**

Asylum seekers and refugees (ASRs) face significant challenges when accessing emergency and pre‐hospital care following injury. Language barriers, unfamiliarity with service pathways and limited trust in authorities hinder access to emergency services. We report results of focus groups with asylum seekers and refugees who have accessed emergency services to understand their use of services and care received.

**Method:**

We recruited participants through community organisations and support networks which advocate and support asylum seekers and refugees across four areas in England and Wales. We gained informed consent to record and transcribe all focus groups. We applied framework analysis to map emergent qualitative themes onto the seven domains of the Candidacy Framework, in order to examine how access to services was recognised, navigated, and negotiated.

**Results:**

We report findings from 35 participants, who demonstrated the ability to recognise situations when emergency care was required, yet their ability to seek help was shaped by uncertainty, fear, and experiences of exclusion. Cultural norms, immigration status, and systemic barriers including complex service pathways and financial constraints affected perceptions of eligibility and influenced help‐seeking after injury. Communication difficulties were the most persistent challenge, cutting across all candidacy domains and compounding existing vulnerabilities.

**Conclusion:**

This study highlights persistent inequities in access to emergency care for asylum seekers and refugees following injury in England and Wales. Using the Candidacy Framework, it demonstrates how language barriers, uncertainty about entitlements, and structural constraints shape how individuals recognise need, navigate services, and experience emergency care. Addressing these challenges requires embedding equity within emergency care systems through improved communication, navigation support, and sensitivity to the lived realities of asylum seekers and refugees.

## 1 Introduction

Injuries constitute a major global public health challenge, causing approximately 4.4 million deaths annually and leaving millions with long‐term disability [3]. In the United Kingdom, unintentional injuries account for around 7 million emergency department (ED) attendances and more than 20,000 deaths each year, generating an estimated £6 billion in annual costs to the National Health Service (NHS) [4]. Despite this substantial burden, relatively little evidence examines how marginalised populations, particularly asylum seekers and refugees (ASRs) experience and access emergency care following injury as global displacement of people continues to rise. According to the UNHCR 2024 mid‐year report, 122.6 million individuals were forcibly displaced worldwide, including 37.9 million refugees and 8 million asylum seekers [5]. While the majority reside in low‐ and middle‐income countries, a significant minority reside in high‐income countries such as the UK.

Asylum seekers and refugees in the UK face complex and intersecting health challenges shaped by pre‐migration trauma and hazardous journeys [6, 7]. ASRs experience disproportionately high rates of physical and mental health conditions including post‐traumatic stress disorder, depression, and cardiovascular disease [6, 7, 8]. Although urgent and emergency care is free at the point of use, under NHS charging regulations ASRs continue to encounter substantial access barriers, including language difficulties, limited familiarity with NHS structures, administrative complexity, restricted entitlements to other forms of care, and concerns about data sharing between healthcare services and immigration authorities [9, 10]. These factors may undermine trust in health institutions and contribute to delayed help‐seeking [6, 7, 8] Notably, these challenges persist despite commitments to reduce health inequalities outlined in the NHS Long Term Plan (2019) [11].

International studies in high‐income countries report elevated rates of injury and trauma‑related ED use among refugee populations, including higher prevalence of unintentional injuries such as motor vehicle collisions, burns, poisoning, and suffocation [12, 13]. These patterns reflect the interaction of individual vulnerabilities with broader structural determinants of health. Emerging evidence suggests that the post‐arrival material conditions characterised by poverty, precarious asylum accommodation, particularly overcrowding, shared facilities, and unsafe living environments, can heighten the risk of unintentional injury along with post‐arrival conditions, and restricted entitlements [14, 15]. Consequently, UK policies governing asylum accommodation, dispersal, and subsistence support could indirectly shape both injury risk and engagement with urgent and emergency care services.

Within the NHS, policy frameworks such as Core20PLUS5 [16], the Urgent and Emergency Care Recovery Plan [17], and commitments to trauma‑informed practice identify inclusion health groups as key priorities for reducing health inequalities. Nevertheless, UK‑specific evidence on injury‑related pathways among ASRs remains limited, and little is known about their emergency care trajectories or interactions with prehospital and ED services [18].

Given these intersecting clinical, structural, and policy challenges, there is a clear need to better understand how asylum seekers and refugees (ASRs) navigate emergency care following injury. This paper reports findings from a qualitative study conducted as part of the wider BE SURE (Building an Understanding of Ethnic Minority People's Service Use Relating to Emergency Care for Injuries) study [1], which investigates ethnic inequalities in injury‐related presentations to emergency services, healthcare experiences, and outcomes. By examining ASRs’ experiences of accessing emergency departments and ambulance services, this study identifies structural and practical barriers to care, with the aim of informing the development of more equitable, culturally responsive, and policy‐aligned emergency care pathways for asylum seekers and refugees in the UK.

### 1.1 Patient or Public Contribution

Patient and public contribution was embedded throughout the BE SURE study [1], ensuring that the perspectives of asylum seekers and refugees directly shaped the research. Two public members, SS and TH, both asylum seekers with lived experience of navigating emergency care after injury, played an integral role as co‐researchers. Their involvement extended across the research cycle, including advising on study materials, supporting data collection, and contributing to the interpretation of emerging findings.

SS and TH participated in regular project meetings, offering critical insights that strengthened the cultural relevance and acceptability of the study approach. Their lived experience enabled the team to refine recruitment strategies, ensure interviews were conducted sensitively, and enhance the depth and authenticity of data interpretation. In addition to shaping analytical discussions, they helped contextualise findings within the broader realities of life as an asylum seeker in the UK, ensuring the study remained grounded in community priorities.

To support meaningful involvement, we provided dedicated points of contact and ongoing pastoral support. SS and TH were offered honoraria and reimbursement for expenses, and all study materials were provided in accessible formats in line with best‐practice public involvement standards [2]. This collaborative approach strengthened both the credibility and the impact of the study, ensuring that BE SURE's insights and recommendations were firmly rooted in the lived experiences of those most affected.

## 2 Method

### 2.1 Conceptual Framework

We used the Candidacy Framework [19] to analyse how individual, sociocultural, and structural factors shape perceptions of eligibility, help‑seeking behaviour, and interactions with emergency services. The framework conceptualises access to healthcare not as a single event but as a dynamic, continually negotiated process, recognising that access is shaped not only by individual need but also by interactions between individuals, healthcare services, and wider structural conditions. It is particularly relevant for research involving vulnerable and socially marginalised populations and is useful where eligibility for care may be uncertain, contested, or constrained by structural factors [20].

The Candidacy Framework outlines seven interconnected domains that trace the journey through which individuals recognise a need for care, navigate services, and experience healthcare responses. Central to the framework is the assumption that candidacy is co‐produced: individuals articulate their need for care, while healthcare systems and professionals assess, negotiate, and respond to that need. This makes the framework particularly well suited to analysing asylum seekers’ and refugees’ encounters with emergency care, where structural exclusion, language barriers, and prior trauma can shape access across the care trajectory.

The framework has previously been applied to asylum seeker and refugee populations. For example, van der Boor and White used the Candidacy Framework to synthesise evidence on access to mental health services among asylum seekers and refugees and found it valuable for understanding how barriers arising from language, service organisation, administrative processes, and social exclusion shape access to care. The authors concluded that the framework provided a useful means of understanding the interrelated barriers experienced when accessing and negotiating healthcare services.

Alternative frameworks were considered, including Levesque et al.‘s Conceptual Framework of Access to Healthcare [21], which we previously applied to examine asylum seekers’ and refugees’ experiences of accessing healthcare in Wales [15]. While that study found the framework useful for identifying barriers and facilitators to healthcare access the Candidacy Framework was considered more appropriate for the present study because it focuses explicitly on how access and eligibility are negotiated between service users and healthcare systems. This emphasis on navigation, gatekeeping, service encounters, and professional adjudication aligns closely with asylum seekers’ and refugees’ experiences of emergency healthcare, where decisions about when, where, and whether care is accessed are often shaped by complex interactions between individual circumstances and healthcare systems.

Applying the Candidacy Framework provided a coherent analytical structure for examining how inequities in emergency care access are produced and maintained, and for identifying potential points for intervention to improve equity.

### 2.2 Setting

Asylum seekers and refugees in the UK are dispersed predominantly across England through the Home Office dispersal system, which allocates asylum accommodation to local authorities with available housing capacity for people claiming asylum [22] while refugees are free to live in any location. England hosts the largest proportion of asylum seekers, with 100,600 individuals included in asylum applications during 2025, the third‑highest annual total on record [23]. In the same period 2025, over 50,000 people were granted refugee status [23]. Home Office regional data shows concentrations of supported asylum seekers in cities such as Sheffield, Leicester, Birmingham, Manchester, and London [24], reflecting long‑standing dispersal patterns.

Wales hosts a smaller but substantial ASR population, including approximately 10,000 refugees, more than 5,600 asylum seekers supported under Section 95/98 arrangements, and over 4000 Ukrainians resettled since 2022 [25, 26]. Although not included as study sites, Scotland and Northern Ireland also accommodate dispersed asylum‐seeking populations. Scotland has participated in the UK dispersal scheme since its inception, with Glasgow remaining one of the largest dispersal locations outside England. Home Office statistics from 2025 indicate that approximately 6600 asylum seekers receiving support were living in Scotland, compared with around 2600 in Northern Ireland, the majority of whom were accommodated through the dispersal system [27]. Together, these figures demonstrate that asylum dispersal is a UK‐wide phenomenon, requiring health and public services across all four nations to respond to the needs of increasingly diverse asylum‐seeking and refugee populations.

### 2.3 Recruitment and Sampling

We used purposive sampling to recruit participants [28] through third‐sector organisations and community groups that support asylum seekers and refugees across three sites in England and one site in Wales in 2024. Participants were purposively selected based on characteristics relevant to the study aims: aged 18 or over, self‐identification as an asylum seeker or refugee (see Table 1), and experience of injury and emergency care in the UK, either directly or through supporting a family member or friend to access care (see Table 2). The inclusion of participants with indirect experience reflected the study's focus on understanding how emergency care is accessed and navigated within asylum‐seeking and refugee communities. Consistent with the Candidacy Framework, healthcare access is understood as a socially negotiated process shaped by interactions between individuals, their social networks, healthcare organisations, and wider structural factors. Family members and community networks often play an important role in decisions about whether, when, and how emergency care is accessed; therefore, accounts of supporting others provided valuable insights into how eligibility for care is understood, negotiated, and enacted in practice.

**Table 1:** Definitions of key terms related to asylum status in the UK.

Term	Definition
Asylum seeker	A person who has applied for asylum in the UK under the 1951 Refugee Convention and is awaiting a decision on their claim.
Refugee	A person whose asylum claim has been accepted and who has been granted leave to remain in the UK due to a well‐founded fear of persecution in their country of origin.
Refused (failed) asylum seeker	A person whose asylum claim has been rejected. They typically have no recourse to public funds and may be liable for removal from the UK, although limited support (e.g. Section 5 support) may be available if they are destitute.

*Note:* Definitions are based on the 1951 Refugee Convention and UK Home Office asylum policy guidance.

**Table 2:** NHS emergency services for injuries in the UK.

Service/component	Description
NHS 111	NHS 111 provides access to urgent care for non‐life‐threatening injuries. Trained advisors offer clinical advice and can refer patients to appropriate services.
Ambulance and hospital care	Patients call 999 or 112 to access ambulance services and hospital emergency departments. Ambulance services provide on‐scene assessment, initial treatment, and transport. Hospital emergency departments have specialist staff and equipment to manage serious and life‐threatening injuries.
Urgent care centres, minor injury units, and GP out‐of‐hours services	These services manage injuries requiring prompt attention but which are not life‐threatening.

*Note:* Based on NHS England ^17^ guidance on urgent and emergency care services.

Focus groups were conducted in four community settings in Surrey, Sheffield, Leicester, and Cardiff. These sites were purposively selected as they were located in Home Office dispersal areas with established asylum‐seeking, refugee, and ethnic minority populations. Consistent with the BE SURE study protocol, which sought to understand ethnic minority experiences of injury‐related emergency care across diverse settings, these locations were chosen to facilitate recruitment from communities most relevant to the study aims. The selected sites also reflected geographical areas served by participating emergency care services within the wider BE SURE study [1].

Selecting sites across three locations in England and one in Wales enabled the inclusion of participants with diverse cultural backgrounds, migration histories, and experiences of healthcare access and service use. Using multiple community settings enhanced the breadth of perspectives captured and allowed exploration of experiences across different local service contexts and support infrastructures. Although healthcare provision and support services vary across regions, many of the barriers identified, including language difficulties, challenges navigating unfamiliar healthcare systems, and uncertainty regarding healthcare entitlements, are likely to be relevant across a range of asylum‐seeking and refugee settings. The findings therefore have applicability beyond the study locations while recognising the influence of local service organisation and community support on participants’ experiences.

To maximise accessibility and inclusion, participant information sheets and consent forms were translated into Arabic, Gujarati, Punjabi, Polish, and Somali, based on local language needs identified by voluntary‐sector partners involved in recruitment. A Ukrainian translation was also produced in response to a participant request. Interpreters supported outreach and communication where required. Recruitment was not limited to these language groups, and participants were drawn from a diverse range of asylum‐seeking and refugee backgrounds. Four focus groups were conducted, each comprising 6–10 participants. Ethical approval for the study was obtained from Swansea University Ethics Committee (reference 2018‐0006) and Health Research Authority (22/WA/0080).

### 2.4 Data Collection

We facilitated focus groups in community settings familiar to participants, aiming to create a safe and comfortable environment. To facilitate discussion and explore pathways to care, we used three injury‐related vignettes, which can help reduce power imbalances in focus group discussions [29, 30]. The vignettes were developed in consultation with research paramedics and emergency care clinicians and were based on common injury presentations to emergency departments. The scenarios involved an older person who had fallen outside her home, a person with a burn injury, and a child who had fallen from a bicycle and had a head injury. These scenarios were selected to reflect a range of everyday injury situations requiring decisions about whether and how to seek emergency care. They were used as discussion prompts to encourage participants to reflect on how they would respond in each situation and to share their own experiences of accessing emergency care.

Each focus group was led by an experienced research team member (AK and AP) and lasted between 60 and 90 min and was conducted in English, with interpreter support in Arabic (in three groups) and Arabic and Ukrainian (one group). We used a semi‐structured topic guide to explore participants’ experiences of injury, decision‐making around seeking care, and barriers to accessing ambulance or ED services. With signed consent from participants, we audio‐recorded all sessions and transcribed them verbatim. Co‐facilitator (FB – Arabic speaker) took field notes during each session to capture non‐verbal cues and contextual observations. Participation was voluntary, and participants received a £20 gift voucher in recognition of their time and contribution.

### 2.5 Analysis

Our analysis followed an inductive theory‐informed approach [31]. Interviews were first analysed using open coding to identify themes emerging directly from the data, guided by the study aims and interview topic guides. The Candidacy Framework was then applied as an analytic lens to the coded data, enabling a systematic examination of barriers and enablers to care for refugees and asylum seekers across the access trajectory [32].

Three qualitative researchers (AK, LJ and HMK) independently read all focus group transcripts and met to discuss initial themes. We then reviewed the data in more depth and organised it into a framework matrix using NVivo qualitative data analysis software (Version 15). NVivo enabled systematic comparison of codes, themes, and transcripts, helping us to identify both consistent and divergent perspectives across participants and to strengthen analytical rigour and inter‐coder reliability [31]. We resolved any differences in interpretation through discussion and re‐reading of the original transcripts and field notes, led by an experienced qualitative researcher (AK), until we reached consensus.

We shared draft findings with members of the Research Management Group (RMG) for critical input. Additional insights from our wider team of researchers, healthcare providers, third‐sector organisations, and public contributors from ASR communities further informed our analysis, particularly in relation to system‐wide and organisational barriers to accessing care.

## 3 Results

We recruited 35 participants to the study, who took part in four focus groups across four areas of the country (see Table 3). Most participants had experience of accessing emergency services, either for their own care or for that of family members and friends. One group comprised men only, one women only, and two were mixed‐gender groups. Participants ranged in age from 19 to 62 years and originated from 17 countries. Most had lived in the UK for fewer than 3 years and were residing in Home Office‐funded temporary accommodation, including hotels, or in dispersal housing. Educational backgrounds varied, ranging from limited formal education to completion of higher education. All participants were either seeking asylum or had recently been granted refugee status.

**Table 3:** Characteristics of participants (*N* = 35).

Characteristic	*n*	%
**Gender**		
Male	16	45.7
Female	19	54.3
**Status**		
Refugee	23	65.7
Asylum Seeker	12	34.3
**Country of origin**		
Afghanistan	2	5.7
Albania	1	2.9
Brazil	1	2.9
Congo	1	2.9
Egypt	4	11.4
Eritrea	2	5.7
Ethiopia	1	2.9
Iran	1	2.9
Iraq	2	5.7
Kurdistan	1	2.9
Namibia	1	2.9
Nigeria	2	5.7
Somalia	1	2.9
Sudan	7	20.0
Syria	4	11.4
Ukraine	1	2.9
Zimbabwe	3	8.6
**Accessed emergency services**		
Personally	19	54.3
For others	14	40.0
Not accessed	2	5.7

We identified key themes across all seven domains of the Candidacy Framework (Table 4). Participants across study sites described complex and often challenging experiences of accessing emergency care, both following injury and for other health needs. Accounts highlighted how cultural beliefs, immigration status, language barriers, and wider systemic factors shaped participants’ ability to seek, negotiate, and receive care.

**Table 4:** Candidacy model applied to emergency service use.

*1. Identification of candidacy*	How individuals interpret symptoms or injuries and decide whether they warrant clinical attention.
*2. Navigation*	The work required to locate, understand, and reach appropriate healthcare, influenced by system complexity, social networks, and health literacy.
*3. Permeability of services*	The extent to which services are accessible and easy to engage with, shaped by organisational structures, administrative processes, and cultural inclusivity.
*4. Appearance at services*	How people present themselves to healthcare providers, including communication ability, confidence, and perceived legitimacy.
*5. Adjudication by professionals*	How healthcare professionals evaluate, validate, or contest an individual's claim to care, often shaped by organisational pressures, implicit bias, and professional norms.
*6. Offers and resistance*	The negotiation of treatment options, where patients may accept, modify, or reject care based on cultural expectations, trust, and prior experiences.
*7. Operating conditions and local Production of candidacy*	The broader socio‑political, economic, and policy environments in which candidacy is shaped, including immigration policies, resource constraints, and service design.

To protect anonymity, excerpts from transcripts quoted are attributed to focus groups rather than individuals [31]. For consistency, findings are presented according to the seven candidacy domains; however, participants’ accounts frequently spanned multiple domains. In particular, issues related to navigation and service permeability were often discussed together, reflecting how lived experiences of access do not align neatly with conceptual boundaries and illustrating the interdependent nature of candidacy processes.

### 3.1 Domain 1. Identification of Candidacy: *Recognising the Need for Care*

Across focus groups, participants generally recognised the need for emergency care when injuries were perceived as severe or life‐threatening. However, many described uncertainty about what constituted a legitimate reason to seek emergency help, even within a healthcare system that is free at the point of use. Decisions to delay or avoid care were shaped by cultural norms around self‐management and stoicism, prior experiences of restricted access to healthcare in countries affected by conflict, financial concerns, and the practical challenges of living in remote or rural areas.

Several participants described past experiences in which distance to healthcare facilities and expectations of self‐reliance influenced decisions about whether to seek care:

Yeah. They don't want [to travel to hospital]– especially from my village [Sudan]. If you have a serious patient emergency situation, you have to drive 1 h or one‐and‐a‐half hour to go to the hospital.

(FG1, Male)

Cultural beliefs and reliance on traditional or home‐based remedies further shaped how participants judged injury severity and the need for professional care:

It's different, different cultures. As he said, like some people they go to like traditional [healer]. It [the treatment] doesn't work but psychologically they like it, it will be okay.

(FG1, Male)

Same like in Afghanistan, some people even get injured in the bomb blasting, they don't go to the doctor… Someone who comes from this background and finds services here difficult to access may not go, because they come from a culture where it's better to deal with things yourself.

(FG1, Male)

Participants also described normalising pain or delaying care even in the presence of potentially serious injuries:

Like sometimes accidents happen and the guy doesn't care. Maybe the bone is broken and they don't care about that, and after weeks and weeks, the pain is coming up and the bone is totally damaged.

(FG 1, Male)

If it's your hand is broken. If you can move it and it's not really painful, you just leave it and let them rest. (FG 2, Female)

When responding to a vignette describing a burn injury caused by hot oil, participants commonly reported attempting self‐management before seeking formal care, with professional help viewed as necessary only if symptoms worsened:

I will first make first aid on myself. And be like cold water or something cold, something make me relax myself, before I pick up my phone, try to call 999… But definitely if I feel like much pain, definitely, ambulance or walk down to the hospital, if they don't come quick, and just walk myself because it is nearby.

(FG 2, Female)

Traditional approaches were also described in the context of managing potentially serious incidents:

I gave my little brother [who took pills in Sudan] milk and he vomit the milk. I don't know what the secret is in the milk.

(FG 1, Male)

For some participants, the identification of candidacy involved weighing perceived injury severity against anticipated waiting times and expectations of care within emergency services. One participant described actively avoiding ambulance conveyance following a road traffic incident due to concerns about delays and the quality of care in emergency departments:

The security guard keep asking me questions and called 999…999 take a long time…I take to my bike and I escaped from there…. But I'll tell you something, here when you go to the ambulance, if you go to A&E, you can stay for long time.

(FG, Male)

Despite these accounts of delayed or avoided care, some participants reflected critically on the potential long‐term consequences of not seeking timely medical attention, particularly for injuries initially perceived as minor:

No, it's not the right way. We have to – if we got even a minor injury, we have to go for check‐up. Not for now, it's – maybe it's not painful, we don't have any issues right now. But maybe ten – after ten years, we get an issue from here. So we have to go to the doctor for a check‐up and to take care.

(FG, Male)

### 3.2 Domain 2. Navigation: System Complexity

Participants consistently described the UK healthcare system as complex and difficult to navigate, particularly when trying to distinguish between different forms of urgent and emergency care. Across all focus groups, participants were familiar with calling 999 to access ambulance services and attending emergency departments (EDs), and many had used NHS 111 for advice and triage. In contrast, awareness of GP out‐of‐hours services, Urgent Care Centres (UCCs), and Minor Injury Units (MIUs) was limited. Those who had encountered these services typically did so through trial and error rather than through clear information or formal signposting.

Although participants did not comment directly on geographical variation in service provision, they frequently emphasised the difficulty of knowing where to seek care and how to access timely support, even when services were advertised as being open until late evening:

They [charity] told us like if your GP's closed, go to walk‐in centre…But they [GP] don't say that in the phone call when you ring them…I came many times to the walk‐in centre., but I mean, it said clearly, I mean, at five they close, here, the walk‐in centre.

(FG 1, Male)

Many participants reported receiving little or no orientation to the healthcare system upon arrival in the UK. As a result, they relied heavily on informal networks, including friends, neighbours, and third‐sector organisations, to navigate emergency care pathways:

I don't know why all the refugees or all asylum seekers, when they arrive in the UK, nobody explains anything, any medical rights … their access to the services.”

(FG 3, Female)

For some, this lack of guidance was especially acute during emergencies shortly after arrival, when unfamiliarity with transport, geography, and basic administrative systems compounded distress:

My son is bleeding, you know, it was raining, and I was afraid, and this happened like almost when we first arrived in the UK, so we don't know about the hospitals, the places. We don't know about the buses, nothing… we don't know even how to order a taxi. I called my friend and they told me, “You can call them [999 ambulance], you give them the postcode. We came from different country. We don't know what is postcode.

(FG 3, Female)

Several participants described learning about services such as NHS 111 only through voluntary or refugee support organisations rather than through statutory services:

I didn't know what 111 was. I only found out from the Refugee Council.

(FG 1, Male)

### 3.3 Domain 3. Permeability: Understanding Emergency Care Pathways and Points of Entry

Navigating emergency care pathways was further complicated by language barriers and the complexity of telephone triage systems. Participants commonly described these processes as burdensome, particularly during periods of acute distress. Emergency call‐taker protocols were perceived as difficult to understand and emotionally challenging, contributing to delays and increased anxiety:

‘When I called the 999, they have been, I mean, asking too many questions. And I have noticed that if I'm not able to understand what they're saying and if you are – when you have someone you care is in a bad situation, you are concentrating. You will not be in a good mood to answer such long questions. I think they have some kind of protocol culture.

(FG 1, Male)

In contrast, NHS 111 was often viewed as more accessible due to the availability of interpreter support and language options, although participants highlighted inconsistencies between services:

111 helped me. Twice I've called them… they can also assess and give you advice. They're like a triage. They'll refer you to 999 and an ambulance will come to you and sometimes it's faster if they can get them for you.

(FG 4, Female)

I mean, when you call 111, if you speak Kurdish, if you speak Arabic, press one, press two. Like something included in system. I wonder why they're not doing it for 999?

(FG 1, Male)

Despite these challenges, emergency services were often perceived as more permeable than primary care, particularly for individuals who were unable to register with a GP due to ongoing immigration processes:

But my daughter just came…She can't get a GP? No, no, any GP, “No, you are not legal…” She can't register… [she can only] register only until they have a paper from the Home Office.

(FG 3, Female)

Participants also drew distinctions between adult and paediatric care, frequently describing children's services as more responsive and accessible:

My son he fall down at school and they called me, “You need to come and pick him up from school.” And I went, I see there is blood and they put things on him and then took him straight away to A&E, children A&E. We sat down about half‐an‐hour and then they called us in and that was it, it was quick.

(FG 2, Female)

[son with epilepsy] …she [call taker] told me that the ambulance is on its way, it will take like a few minutes. The impressive thing regarding this ambulance came very quick. I really felt grateful for this, and they started to keep me calm down a little bit. Then when we went to the hospital, I found the hospital team was like waiting, waiting for us.

(FG 3, Female)

Comparisons with healthcare access in countries of origin or transit were common, with participants often describing systems elsewhere as more straightforward and locally available, despite being privately funded:

That's the difference between here and our country [Syria]. Our country, we didn't used to go to local hospital. All the time, we go to private, because it's cheaper. Here, if you want to go to private, you have to pay a lot of money.

(FG 3, Female)

“when we were in Turkey [as refugees], every place, there is emergency point. It's close to everyone. Anyone can go there. No need to call, no need to do anything. Here, if there is emergency situation happened, you have to call. And even if you have a [hospital] point close to you, you have to go – you have to travel sometimes, and it's very far.

(FG 3, Male)

Finally, participants highlighted how immigration status and accommodation arrangements shaped entitlement to practical support, particularly transport costs when accessing emergency care. These inconsistencies were seen as distressing and as potential deterrents to future care‐seeking:

If they [migrants] live in hotel they provide taxi to come back. When they move to the house they don't. They took me to the hospital by ambulance. And like 3 or 4 o'clock, they told me that, “You have to go at midnight.” I don't have money [to return home] and when I called to my person responsible, the company. They said, “We don't provide taxis for who live in accommodation.” That time, the taxi charge thirty‐five pounds. I have only forty pound per week for my food. So how can I manage?

(FG 1, Male)

Several participants were unaware of entitlement to support through the NHS Low Income Scheme (HC2 for full help or HC3 for partial help) [32] and described covering travel costs themselves while awaiting documentation:

If you use your money to go to hospital, you need to claim it back. But I never claimed it back, until I got my papers.

(FG 2, Female)

### 3.4 Domain 4. Appearance at Emergency Services: Presenting Need and Asserting Candidacy

Participants frequently described anxiety about attending emergency departments or contacting ambulance services, with language barriers intensifying difficulties in articulating symptoms and responding to questions during moments of acute distress.

Several participants reported being unable to communicate effectively with call handlers and described not being offered interpreter support, limiting their capacity to present their need for care clearly:

I was not able to talk with him [call handler] correctly, because I was in a very bad situation. But they didn't ask me for interpreter and anything else. Just they asked a few questions and I ask again and again, “What do you mean? Can you explain all that?”

(FG 1, Male)

In emergency situations involving children, difficulties in communication heightened fear and urgency, with participants describing how their inability to respond appropriately to repeated questions delayed assistance:

They kept asking me same questions, and I felt I couldn't be able to answer these questions and I can't even recognise what you are asking about. So, then while asking me, my son's skin turning blue, so then I started to shout, “Please come now.”

(FG 3, Female)

Perceived delays and the complexity of telephone triage processes shaped how participants chose to present to services. Some reported deciding to self‐present at hospital rather than rely on ambulance services, particularly when response times were perceived to be excessively long:

my [friends] child hurt himself and we didn't know whether to take him out [to the ED] or wait – the ambulance told me it will take 6 h for them to come out. So we had, I had to call another friend to take him to hospital.

(FG 3, Male)

These accounts illustrate how barriers to communication and expectations surrounding how emergencies should be presented influenced participants’ ability to assert their candidacy and access timely care.

### 3.5 Domain 5. Adjudications: Service Responses and Judgements of Eligibility

The *adjudications* domain captures how healthcare professionals interpret patients’ presentations and determine eligibility, urgency, and appropriate response. Participants described instances where their mode of arrival, communication style, or perceived credibility influenced how seriously their health concerns were taken.

One participant described presenting to hospital with chest pain but feeling that staff dismissed the seriousness of his condition because he had arrived on foot rather than by ambulance:

[I don't call] 999, they aren't coming. I went to the hospital [self presented], because I needed to be seen [with chest pain]. And they're just asking, “How did you come here?” I said, “Like just walking.” And they think that I'm okay because I came here walking. So to be honest, you know, I wasn't okay.

(FG 1, Male)

Another participant linked an inadequate clinical assessment to long‐term health consequences, interpreting the adjudication as insufficiently thorough:

My son he had a fall on his nose. So I really wanted Dr to do an X‐ray for him because, you know because he [son] looks blue are not happy with it. But he [Dr] just done like – just like touched him [brief checkup] and that's it. “He's okay. He's fine, it's not broken.” After, It's been like 2 years, [Son] struggling with breathing. He had lots of nose bleeds. We went to – for a different country [Turkey], we paid private. We found out that his nose was broken and it's really bad broken.

(FG 2, Female)

Fear of being judged or reported to authorities further shaped experiences of adjudication, particularly for parents who worried that seeking care could trigger safeguarding scrutiny linked to their migrant status:

the first question was, “Do you have a social worker?” maybe because she knew I'm an asylum seeker, and I told my husband, “Next time, if anything happens like this, I will not call them, because maybe they will make a problem.

(FG 3, Female)

Participants contrasted these experiences with practices in their countries of origin, describing the need to adapt to unfamiliar expectations about parental behaviour and interaction with health professionals:

“Back home, you do whatever it takes to help your child. Nobody will say “we see your hands here touching the child, you were harming the child”, Here, you've got to let NHS take care of it… I think we have to adopt the culture of where we are.

(FG 2, Female)

Some participants perceived questioning by ambulance staff as intrusive and reminiscent of immigration processes, reinforcing feelings of surveillance and mistrust:

Even the ambulance crew they're really disappointing me at one time, they asked questions like the Home Office.

(FG4, Female)

Others described differential treatment in community and hospital settings, which they attributed to their migrant status:

I think they really treat you differently [migrant status]. They tried to take his tooth out in the dentist with a doctor. He was bleeding and they sent him home with this bleeding. And he tried to get to the A&E, but there they said, “We can't do nothing. He went back again to the dentist and then they said, “We can't do anything.” He ended up calling police because they [dentist] don't even open the door for him.

(FG4, Male)

Despite these negative experiences, participants also reported positive encounters with individual healthcare professionals, emphasising that their concerns were often directed at systemic processes rather than frontline staff:

Yeah, it was – the ambulance guy treat me very good. And every 5 min they asked me, “Are you okay?” They are very nice people. They are cooperating, they're being really nice to you. It's not about the care, it's about, I think, the system.

(FG3 Male)

### 3.6 Domain 6. Offers and Resistance: Interplay Between Emergency Service Provision and Uptake of Care

Across focus groups, participants commonly described resistance to the offer of care, particularly in relation to the 999 ambulance service. Prior negative experiences with ambulance services, and in some cases NHS 111, shaped decisions to avoid phone‐based access routes altogether. Many participants reported preferring to travel directly to hospital, even in emergency situations, to avoid the stress of lengthy triage processes conducted in English and without consistent language support. Difficulties communicating with call handlers and ambulance staff were described as confusing and distressing, while anticipated delays in ambulance response further discouraged help‐seeking.

I would say we never call 111 or 999 because it will take a long time.

(FG 3, Female)

“I had no bus money, and the hospital was far. I waited until it got worse.”

(FG 3, Male)

In contrast, a minority of participants perceived ambulance conveyance as advantageous, believing that arrival by ambulance resulted in shorter waiting times in the ED:

I go in an ambulance it's less time [waiting in ED], it's much better. If you go by yourself [to ED], you wait longer.

(FG 4, Female)

Concerns about language support featured prominently in accounts of resistance to care uptake. Participants frequently raised issues regarding the limited availability, quality, and consistency of interpreters during emergency care encounters. Where interpretation was provided, participants often questioned its accuracy, leading to mistrust, misunderstanding, and reluctance to re‐engage with services. Inadequate communication was perceived to pose risks to patient safety, particularly around medication use, and reinforced reliance on informal networks for advice and clarification:

The interpreter didn't explain properly. I didn't trust what was being said.

(FG 2, Female)

‘They write in English [information on how to take medication] and nobody explained for them that you have to take [medication] like this. I have met someone like they went to the doctor, they say, “Go take the medication.” They don't know from where I have to collect the medication. One guy, the GP give him like a tablet for stomach and told him that you have to take one in a day. He took three in a day, because of no interpreter.

(FG 1, Male)

These experiences illustrate how the perceived offer of care, shaped by communication barriers and prior encounters, directly influenced participants’ willingness to accept or resist engagement with emergency services.

### 3.7 Domain 7. Operating Conditions and Local Contingencies: Contextual Factors

The final candidacy domain captures how operational conditions and local contextual factors shape access to and experiences of emergency care. Participants described how factors such as staffing pressures, adherence to rigid protocols, and limited flexibility within emergency systems influenced their interactions with services. Communication barriers further amplified frustration, particularly during telephone triage, where questioning was experienced as impersonal and distressing during acute health crises:

“Are you still breathing?” I was so mad because you can hear how I was crying, falling on the floor, can't stand up. I was in serious pain and the guy [call taker] keep asking, “Are you still breathing? Can you walk down stairs?” Like there are some questions [triage questions] that they have that I think it's [should] not supposed to be part of the questionnaire.

(FG 4, Female)

Some participants interpreted strict adherence to protocol as a lack of empathy, contrasting procedural requirements with expectations of humane care:

But the system I mean, they follow the system very strictly, some of them [NHS staff]. Some of them are not applying…to human sometimes.

(FG 1, Male)

Across focus groups, participants called for clearer communication, improved availability of high‐quality interpreters, and better orientation for newcomers on how to access healthcare services. Many emphasised the importance of community‐based education and culturally sensitive, multilingual resources to support understanding of rights and navigation of emergency care pathways. Practical barriers, including distance to hospital and transport costs, were repeatedly identified as contributors to delayed presentation following injury.

Some participants also expressed perceptions that immigration status influenced the quality of care received. One individual associated free access to healthcare with lower standards of care, drawing on comparisons with healthcare systems in their country of origin:

to be honest, because I was thinking like, because I'm an asylum seeker and I got treatment free. So, for this reason, they are treating me like this [long wait times].

(FG 1, Male)

The accounts presented here highlight how operating conditions, local contingencies, and broader structural factors interact to shape experiences of emergency care, influencing both perceptions of care quality and decisions about when and how to seek help, particularly in contexts of increasing numbers of asylum seekers and refugees in the United Kingdom.

## 4 Discussion

### 4.1 Summary of Key Findings

This study provides an in‐depth examination of how asylum seekers and refugees (ASRs) navigate emergency care following injury, using the Candidacy Framework to demonstrate how access is actively negotiated across interactions between individuals, services, and wider structural conditions [19, 20]. While many of the barriers identified in this study have been reported previously, application of the Candidacy Framework advances understanding by demonstrating how these barriers operate across the entire emergency care pathway rather than as isolated obstacles. Previous studies have largely described individual barriers such as language difficulties, limited knowledge of services, or uncertainty regarding entitlement [9, 10]. By contrast, the candidacy lens illustrates how these factors interact and accumulate over time, influencing how asylum seekers and refugees recognise their need for care, judge their eligibility, navigate services, communicate with providers, and engage with treatment recommendations. Access to emergency care therefore emerged as an ongoing process of negotiation rather than a single decision or event.

Across all seven domains of candidacy, participants described how uncertainty about entitlements and the wider asylum system constrained their ability to recognise need, navigate services, assert eligibility, and take up offers of care. Communication difficulties cut across these processes, shaping help‐seeking decisions, navigation of services, and interactions with emergency call handlers and clinicians [33, 34].

Importantly, barriers to emergency care did not arise solely at the point of clinical encounter. Rather, participants’ accounts illustrate the cumulative effects of precarious living conditions, financial hardship, transport insecurity, and repeated relocation within the asylum system [35, 36, 37]. These findings structurally mirror previous work highlighting how asylum policies and housing arrangements directly influence health system access and equity [9, 10, 15].

Together, the findings show that access to emergency care is not a single event but an ongoing process of negotiation. Difficulties encountered at earlier stages of candidacy frequently compounded later barriers, shaping expectations of care and future help‐seeking behaviour [20, 21].

### 4.2 Communication Barriers and Candidacy Negotiation

Consistent with previous research, language barriers operated as a pervasive constraint across all seven domains of candidacy [33, 34]. Difficulties communicating with emergency call handlers, ambulance staff, and clinicians limited participants’ ability to articulate urgency, understand advice, and engage confidently with services. Reports of delayed ambulance response and complex triage questioning conducted in English, often without interpreter support, were experienced as distressing and contributed to mistrust and avoidance of emergency services [33, 34].

These findings reinforce wider literature demonstrating that inadequate access to professional interpreters compromises patient safety, delays care, and increases the risk of inappropriate or harmful treatment [33, 34, 38]. Communication access should therefore be conceptualised as integral to emergency care delivery, rather than as an optional adjunct, if equity and safety are to be achieved [38, 39].

### 4.3 Navigation, Permeability, and Adjudication Within Emergency Care

The Navigation and Permeability domains highlighted how limited orientation to the UK healthcare system left many participants reliant on informal networks and third‐sector organisations for guidance. While such networks often facilitated access, their uneven availability reinforced inequity and placed a substantial burden on third‐sector organisations [9, 15]. Similar challenges in navigating urgent and emergency care have been widely reported among ASRs in the UK, particularly during early stages of settlement [9, 10].

Within the Adjudications domain, participants described concerns about being judged or misunderstood, particularly in relation to immigration status, mode of arrival, and parenting practices. Such perceptions, regardless of individual clinician intent, shaped expectations of care and discouraged future help‐seeking, echoing analyses of how professional judgements of eligibility and credibility are socially mediated and institutionally situated [18, 40]. Fear of safeguarding scrutiny or negative assumptions further reinforced inequities at the point where emergency care decisions are made [18].

### 4.4 Structural Constraints and Operating Conditions

Structural factors linked to the asylum system, including accommodation arrangements, repeated relocations, documentation delays, and financial precarity, emerged as persistent constraints on emergency care access. The Candidacy Framework demonstrates that these barriers were not solely attributable to individual characteristics, but arose through interactions between personal circumstances, service design, and wider asylum policies. Participants’ experiences suggest that opportunities to access care were shaped by the material conditions of seeking asylum, shifting attention from individual explanations towards the organisational and structural factors that influence healthcare access. These findings align with evidence demonstrating how immigration and housing policies can undermine healthcare access by shaping the conditions under which care can realistically be sought and reached [35, 36, 37].

Operating conditions within emergency services, including rigid adherence to protocols, time pressures, and limited flexibility during distress, were often interpreted as impersonal or lacking empathy, particularly during telephone triage. While participants frequently emphasised positive interactions with individual staff, frustrations were directed at the system, underscoring the need to locate responsibility for inequitable access within service design rather than frontline practice alone. Similar observations have been reported by emergency care providers, who describe tensions between institutional demands and the provision of responsive, person‐centred care for ASRs [18].

### 4.5 Comparison With Existing Evidence

Our findings are consistent with UK and international research demonstrating that asylum seekers and refugees (ASRs) face multiple barriers to seeking healthcare access and often delay or avoid care due to language difficulties, uncertainty regarding healthcare entitlements, transport costs, fear of discrimination, and limited familiarity with healthcare systems [9, 10, 15, 33, 34, 36]. Similar patterns of delayed help‐seeking and crisis‐driven use of emergency services have been reported in other European contexts, where barriers to primary care and limited understanding of healthcare pathways contribute to greater reliance on emergency departments [10, 18].

While many of these barriers have been described previously, applying the Candidacy Framework enabled us to move beyond identifying barriers to understanding how access is negotiated between individuals and healthcare services. The findings suggest that barriers are interconnected and cumulative rather than discrete. Uncertainty regarding entitlements influenced help‐seeking decisions and navigation of services, communication difficulties shaped interactions with providers and perceptions of eligibility, and structural constraints associated with the asylum system affected opportunities to seek and reach care. This highlights how inequities in access are produced through interactions between social, organisational, and policy factors rather than through individual behaviour alone [19, 20].

Many of the challenges identified are not unique to ASRs and have also been reported among ethnic minority and other underserved populations, including difficulties navigating healthcare systems, communication barriers, experiences of discrimination, and reduced trust in healthcare institutions [41, 42, 43]. However, the circumstances of seeking asylum may intensify these challenges through the combined effects of insecure housing, financial hardship, displacement, trauma, and uncertainty regarding healthcare rights and entitlements [34, 35, 36]. Participants frequently relied on family members, friends, community organisations, accommodation staff, and faith groups to interpret symptoms, facilitate communication, and navigate care pathways. Similar findings have been reported internationally, where trusted community networks play a key role in overcoming barriers to healthcare access among refugee populations [9, 44].

Importantly, these findings challenge explanations that frame patterns of emergency care use primarily as a consequence of individual knowledge deficits or inappropriate healthcare‐seeking behaviour. Such deficit‐based perspectives risk locating responsibility for poor access within individuals and communities, while overlooking the organisational and structural conditions that shape opportunities for care [45, 46]. In contrast, participants demonstrated considerable agency and resourcefulness in navigating complex healthcare systems despite linguistic, financial, and social constraints. They actively sought advice, drew on community networks, used informal interpretation support, and developed strategies to overcome barriers to care. Viewed through an asset‐based perspective, these findings highlight the strengths, resilience, and social resources mobilised by ASRs when accessing healthcare [46, 47].

Taken together, the findings suggest that emergency care use among ASRs should be understood less as a consequence of individual deficits and more as an adaptive response to constrained access opportunities. Consistent with the Candidacy Framework, healthcare seeking emerged as a negotiated process shaped by interactions between individuals, healthcare systems, community networks, and wider asylum policies [19, 20]. These findings highlight the importance of addressing structural barriers while recognising and building on existing community assets to improve equitable access to emergency care.

### 4.6 Strengths and Limitations

A key strength of this study lies in its application of the Candidacy Framework, which enabled a nuanced analysis of access to emergency care that captures interactions across individual, organisational, and structural levels [19, 20]. The inclusion of participants from multiple sites in England and Wales enhanced the diversity of experiences captured and included the perspectives of individuals who had both accessed and avoided emergency care services.

However, we acknowledge several limitations. There is a possibility of participant bias, as participants were largely recruited by third sector organisations and may therefore reflect individuals who were more socially connected or engaged with support services. Participants taking part in the study came from a range of countries of origin and cultural backgrounds, reflecting the diversity of asylum‐seeking and refugee populations in the UK. Although experiences may vary according to cultural norms, migration histories, and previous healthcare systems, this study did not seek to compare experiences between specific national or ethnic groups. Rather, it aimed to identify common experiences of accessing and navigating emergency care among people seeking asylum or refuge in the UK. Despite their diverse backgrounds, participants described similar barriers, including language difficulties, limited familiarity with healthcare services, and uncertainty regarding healthcare entitlements. Nevertheless, cultural and contextual factors may influence how such barriers are experienced and navigated, and further research exploring differences between specific asylum‐seeking and refugee groups would be valuable. As with all qualitative research, the findings are contextually situated; however, they are consistent with wider national and international evidence on healthcare access among asylum seekers and refugees [9, 10, 36].

### 4.7 Implications for Practice and Policy

The findings suggest several areas that may warrant consideration across multiple candidacy domains.

Rather than focusing solely on changing individual knowledge or behaviour, they highlight opportunities to improve how healthcare systems accommodate the needs and circumstances of refugees and asylum seekers.

Communication barriers emerged throughout the care pathway, suggesting that consistent access to professional interpretation remains a fundamental component of equitable emergency care [35, 48]. Participants’ experiences indicate that system‐level measures, such as routinely incorporating interpreter needs into triage, call‐handling and referral processes, may help reduce reliance on patients requesting support themselves and minimise unintended barriers to access. Likewise, ensuring that information about emergency and urgent care services is available in multiple languages and accessible formats may support confidence and informed decision‐making.

Participants also described uncertainty regarding healthcare pathways and entitlement to services. While orientation and information provision may be helpful, the findings suggest that access difficulties cannot be addressed through education alone. Consistent with asset‐based approaches to health improvement [45, 46], information should build upon existing community knowledge and networks rather than assume a lack of capability among asylum seekers and refugees. Community organisations, refugee support groups, faith organisations and accommodation providers often acted as trusted sources of advice and reassurance, suggesting that they may represent important partners in promoting equitable access to care.

More fundamentally, participants’ accounts point towards the importance of service design that recognises the realities of seeking asylum. Examples include improving access to interpretation across the patient journey, simplifying registration and administrative processes, ensuring flexibility for individuals experiencing housing instability or transport insecurity, and improving communication between statutory services and third‐sector organisations. Such changes seek to reduce structural barriers within healthcare systems rather than placing responsibility for overcoming these barriers on individuals [15].

The findings also point towards the potential value of community navigation and advocacy approaches. Community navigators, community health workers, cultural brokers and link workers have been used increasingly within migrant and refugee health programmes to support service navigation, communication and engagement with healthcare providers [15]. Recent evidence suggests that these approaches can improve healthcare access, trust and system navigation among underserved populations by providing culturally and linguistically appropriate support and by helping individuals negotiate complex healthcare systems [20, 21, 44, 47]. Importantly, such models do not replace mainstream services but seek to bridge gaps between communities and healthcare providers.

However, the present study was not designed to evaluate specific interventions. Rather than identifying particular solutions, it highlights mechanisms through which inequities in emergency care access may arise and identifies areas where system‐level changes may merit further consideration. Future research should examine whether approaches such as enhanced interpretation provision, community navigation support, co‐produced information resources, organisational flexibility and stronger partnerships between healthcare and community organisations are effective in reducing barriers to emergency care for refugees and asylum seekers.

Improved orientation to emergency care pathways for newly arrived ASRs, delivered through trusted community and accommodation settings, may support navigation, reduce anxiety, and avoidable delays [9, 10, 15]. Community‐based education on injury prevention, self‐management, and rights to care may further support informed decision‐making while avoiding deficit‐based approaches [19, 39]. Beyond individual training for those providing and seeking care, findings point to the importance of system design that recognises the realities of asylum of the material constraints of asylum, including housing instability and transport insecurity, to ensure equitable access to emergency care [36, 37, 38].

## 5 Conclusion

This study advances understanding of emergency care access for asylum seekers and refugees by demonstrating how barriers operate cumulatively across multiple stages of candidacy. Rather than reflecting isolated problems of language, knowledge, or service use, access emerged as a negotiated and relational process shaped by interactions between individuals, healthcare organisations, and the wider asylum system. Applying the Candidacy Framework enabled us to move beyond simply identifying barriers to understanding how eligibility for care is recognised, negotiated, and enacted in practice, and how interactions with emergency care services can reinforce or reduce inequities.

The findings highlight persistent inequities in access to emergency care following injury in England and Wales. Language barriers, uncertainty regarding healthcare entitlements, and structural constraints associated with the asylum system influenced how participants recognised need, navigated services, communicated with providers, and experienced care. By demonstrating how these factors interact across the care pathway, this study shows that inequities arise not solely from individual circumstances but from the combined effects of organisational processes, service design, and wider social and policy contexts.

Achieving equity in emergency care therefore requires a system‐level approach that extends beyond individual behaviour change. This includes routine access to professional interpretation, culturally responsive and trauma‐informed practices, enhanced navigation support, and service designs that recognise the realities of seeking asylum, including housing instability, financial insecurity, and frequent relocation. Addressing these wider determinants of access is essential if emergency care services are to respond equitably to the needs of asylum seekers and refugees.

## Author Contributions

**Ashrafunnesa Khanom:** conceptualisation, investigation, funding acquisition, writing – original draft, methodology, writing – review and editing, formal analysis, project administration, supervision, data curation, resources. **Lydia Jones:** formal analysis, data curation, writing – review and editing. **Hanifah M Khanom:** formal analysis, data curation, writing – review and editing. **Fadi Baghdadi:** writing – review and editing, formal analysis, data curation. **Ann John:** writing – review and editing, funding acquisition. **Alison Porter:** funding acquisition, writing – review and editing. **Helen Snooks:** funding acquisition, writing – review and editing, investigation, conceptualisation, methodology. **Niroshan Siriwardena:** funding acquisition, writing – review and editing, methodology. **Julia Williams:** funding acquisition, writing – review and editing. **Thanuja Hettiarachchi:** writing – review and editing. **Solmaz Safari:** writing – review and editing.

6

Funding was received from the NIHR Health and Social Care Services Delivery Research (HS& DR) award NIHR132744.

## Ethics Statement

We received approval from the Health Research Authority (22/WA/0080) and Swansea University Medical School Ethics Committee (reference 2018‐0006).

## Conflicts of Interest

The authors declare no conflicts of interest.

## Data Availability

The data that support the findings of this study are available on request from the corresponding author. The data are not publicly available due to privacy or ethical restrictions.
